# Recruitment and Participation of Recreational Runners in a Large Epidemiological and Genetic Research Study: Retrospective Data Analysis

**DOI:** 10.2196/resprot.8243

**Published:** 2018-05-23

**Authors:** Silvia Manzanero, Maria Kozlovskaia, Nicole Vlahovich, David C Hughes

**Affiliations:** ^1^ Australian Institute of Sport Department of Sports Medicine Australian Sports Commission Bruce Australia; ^2^ Research Institute for Sport and Exercise University of Canberra Bruce Australia; ^3^ Faculty of Health Sciences and Medicine Bond University Robina Australia

**Keywords:** genetic research, community participation, epidemiologic methods, informed consent

## Abstract

**Background:**

With the increasing capacity for remote collection of both data and samples for medical research, a thorough assessment is needed to determine the association of population characteristics and recruitment methodologies with response rates.

**Objective:**

The aim of this research was to assess population representativeness in a two-stage study of health and injury in recreational runners, which consisted of an epidemiological arm and genetic analysis.

**Methods:**

The cost and success of various classical and internet-based methods were analyzed, and demographic representativeness was assessed for recruitment to the epidemiological survey, reported willingness to participate in the genetic arm of the study, actual participation, sample return, and approval for biobank storage.

**Results:**

A total of 4965 valid responses were received, of which 1664 were deemed eligible for genetic analysis. Younger age showed a negative association with initial recruitment rate, expressed willingness to participate in genetic analysis, and actual participation. Additionally, female sex was associated with higher initial recruitment rates, and ethnic origin impacted willingness to participate in the genetic analysis (all *P*<.001).

**Conclusions:**

The sharp decline in retention through the different stages of the study in young respondents suggests the necessity to develop specific recruitment and retention strategies when investigating a young, physically active population.

## Introduction

Large-scale recruitment for research studies is now more easily achieved because of the internet [1]. In addition, new technologies simplify genetic research by enabling unassisted sample collection in the participant’s own home [2]. However, when compared with the traditional recruitment methods, the distance imposed by this methodology challenges sampling bias as well as the ability to provide individualized information to participants for informed consent. Additionally, this method may hinder the development of trust, which according to some authors aids in recruitment to genetics studies [3,4].

### Internet-Based Recruitment for Research Studies

Internet-based sample collection has become commonplace for health research studies. For example, a recent systematic review covered 110 studies that used Facebook to recruit up to 12,000 adult participants per study [5]. Given this trend, it has been recommended that recruitment strategies in research studies are evaluated and reported [6]. Overall, recruitment analysis conducted to date has assessed studies designed to evaluate specific health conditions or the general population, and some authors have studied the association between participants’ physical activity and recruitment success [3]. However we are not aware of any research studies that have analyzed recruitment success and the efficacy of different strategies to recruit participants from a physically active population.

### Participants’ Attitudes to Genetic Data Collection

Clinical data belong to an individual’s sensitive personal information, and genetic data pose specific ethical and security concerns for participation in research projects [7]. Studies show that the public appears to have a positive view of genetic research; however, this may not be associated with actual willingness to participate in a genetic study [8,9]. Demographic factors such as sex, age, education, and ethnicity have shown varying degrees of association with declared willingness to participate or actual participation in genetic research [10,11]. The informed consent process needs to address the concern for privacy intrusion by presenting information to participants in a clear and simple way [7], and the potential obstacles of Web-based contact in this regard warrant a specific evaluation [9]. All of these factors need to be reported to improve our management of research studies [12].

To our knowledge, the AIS (Australian Institute of Sport) Running Injury Study is the largest genetic study to date conducted on a physically active cohort [13,14]. The analysis presented here had two aims: (1) to assess the success and demographic representativeness of different recruitment strategies and (2) to highlight subject’s characteristics associated with declared willingness to participate and with actual participation in the genetic arm of the study.

## Methods

### Overview

The AIS Running Injury Study was an initiative of the AIS and the Collaborative Research Network for Advancing Exercise and Sport Science in response to the dramatic increase in recreational running in Australia in the past decade [13,14]. The study was approved by the Bond University Human Research Ethics Committee (approval RO1688B). The initial aim was to recruit 10,000 participants and the goal was two-fold: (1) to document health profiles and injury rates in recreational runners, (2) to discover gene-environment interactions associated with two common types of lower leg injuries in runners—Achilles tendinopathy and bone stress injury. The survey, available online through the SurveyGizmo platform (Boulder, CO, USA) for a period of 25 months, played two roles: (1) to measure self-reported running habits, injury profiles, physical and mental health indicators, and nutrition in recreational runners and (2) to act as a screening tool by revealing factors for eligibility to the genetic study.

The genetic study involved an email request to eligible participants to confirm their postal address containing an electronic copy of the participant information sheet and consent form. Email respondents were mailed information and consent documents to sign, a saliva collection kit (Oragene DNA Collection Kit, DNA Genotek Inc., Ontario, Canada), instructions for collection, and a reply-paid envelope. The consent form included a request for permission to store the participant’s sample in an approved Sports Science & Exercise Biobank specimen repository and shared with other ethically approved research teams. Participants were deemed unreachable when no response was received to the initial email plus 2 reminders, or when their sample was not returned after delivery plus 2 email reminders. A second saliva kit was sent to a small number of those who reported lost or damaged kits.

### Participants and Survey

Participants self-selected for inclusion in the survey on the basis of the following criteria: recreational runners aged 18 or older who run more than 15 km per week. Between September 2014 and October 2016, 9069 participants initiated the 30-min Web-based survey and 5248 completed it. Some participants completed the survey 2 times (N=233), some 3 times (N=13), 4 times (N=3), or 5 times (N=1). Only the most recent submission was used for data analysis. Despite the selection criteria, 213 participants reported running less than 15 km per week in the actual survey, but a decision was made to include them in the current analysis.

Eligibility for genetic analysis was complex and participants were not made aware of inclusion criteria so they would not self-deselect from the survey on this account. It included the following: running more than 15 km per week, 18 to 50 years old, having selected the items “Caucasian European” or “Mediterranean” for at least 3 grandparents when asked about their ethnicity, nonsmoker, absence of musculoskeletal comorbidities (arthritis or osteoporosis), medications (quinolone antibiotics, chemotherapy, or others), and recent lower limb fractures. Ethnicity was restricted to simplify genetic association analysis, which in multiethnic cohorts can potentially conceal or confound weak genetic effects [15]. An Australian address was required to eliminate issues with import of biological material. A key requirement for eligibility was acceptance of the following: “I give permission, if I am eligible, to be contacted in the future to provide a saliva sample for genetic related analysis.” No individual feedback was promised to participants at any stage of the study.

### Procedure

The full panel of recruitment activities used throughout the study is presented in Table 1. To assess if this cohort was representative of the Australian population of runners, the sample was compared with the 2016 AusPlay survey, based on telephonic interviews of a probability-based sample of 70,000 [16]. In January 2016, with only 2232 respondents of the targeted 10,000, a survey item was added asking respondents how they had learned about the study.

**Table 1:** Summary of the recruitment strategies used for this study including the outcomes obtained other than recruitment. The total cost per strategy and the cost per participant in Australian dollars are estimated.

Strategy, methods, and channels		Outcomes (other than recruitment)	Types of expenses	Estimated cost (Aus $)	Participants (n)	Cost per participant (Aus $)
**Facebook**							
	Group page with regular posts	Page followers and post sharing	N/A^a^	N/A	N/A	N/A
	Paid advertisements	N/A	N/A	N/A	N/A	N/A
	Posts in other pages	N/A	Advertising fees	1624	979	1.66
**Other social media**							
	Twitter	N/A	N/A	N/A	N/A	N/A
	Instagram	N/A	N/A	N/A	N/A	N/A
	Newsletters	N/A	None	0	324	0
**Web-based media**							
	Relevant articles	Presence in webpages	N/A	N/A	N/A	N/A
	Radio interview	Podcast	N/A	N/A	N/A	N/A
	Press interview	Blog post	None	0	161	0
**Running events**							
	Flyers, emails to event participants	N/A	N/A	N/A	N/A	N/A
	Presence in race results emails	N/A	N/A	N/A	N/A	N/A
	Contact with running-related businesses	Further promotions	Travel	4173	458	9.11
**CityFit Expo**							
	Flyers	N/A	Printed materials, travel, stand booking and fitting	N/A	N/A	N/A
	Presence in Expo social media	N/A	Printed materials, travel, stand booking and fitting	N/A	N/A	N/A
	Contact with running-related businesses	Further promotions	Printed materials, travel, stand booking and fitting	5249	160	32.81
**Parkrun**							
	Presence at events	N/A	N/A	N/A	N/A	N/A
	Newsletters	N/A	N/A	N/A	N/A	N/A
	Facebook	N/A	None	0	368	0
**Referrals (personal and professional)**							
	Emails to previous survey participants	Facebook posts	N/A	N/A	N/A	N/A
	Sports health professionals	Advice to patients	N/A	N/A	N/A	N/A
	Word of mouth	N/A	None	0	144	0
**AIS^b^**							
	Website	N/A	N/A	N/A	N/A	N/A
	Social media	N/A	None	0	133	0
**Email**							
	Running events	Mentions in newsletters	N/A	N/A	N/A	N/A
	Running clubs	Invitations to events	N/A	N/A	N/A	N/A
	Running-related businesses	Facebook, other social media	N/A	N/A	N/A	N/A
	Fitness business, personal trainers, running coaches	Referrals	N/A	N/A	N/A	N/A
	Triathlon and athletics state organisations	N/A	None	0	Unknown	0
**Incentives**							
	Discount promo codes to participants	N/A	N/A	N/A	N/A	N/A
	Competitions	N/A	None	0	Unknown	0
Total				11,046	2760	4

^a^N/A: not applicable.

^b^AIS: Australian Institute of Sport.

The purpose was to monitor the success of each recruitment channel with the goal of optimizing future efforts. Data from the remaining 2776 responses were analyzed based on this item. Finally, the demographic factors associated with participants’ willingness to be contacted for genetic research, their actual participation in the research once contacted, and their consent to have their genetic material stored in a biobank were assessed.

### Statistical Analyses

Categorical data were arranged in contingency tables and assessed by goodness-of-fit chi-square test in which the expected frequencies (E) were determined from the observed frequencies and tested with the formula (O-E)^2^/E, except where stated otherwise. The null hypothesis was that the observed frequencies did not differ from the expected frequencies and it was rejected if *P* value was <.05. Cells for which the standardized residuals had an absolute value higher than 5.00 were considered major contributors to the statistically significant chi-square value and indicated with asterisks. All statistical analyses were conducted using the R statistical software package (R Core Team) [17].

## Results

### Aim 1: The Recruitment Process

**Figure 1:**
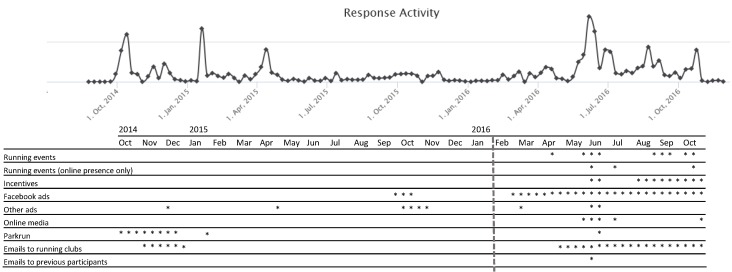
Response activity during the recruitment period. The vertical dotted line at the end of Jan 2016 represents the date at which the additional survey item was included to determine the recruitment channel reported by respondents.

Age, sex, country of citizenship, and weekly running distance are associated with recruitment strategy. Recruitment strategies spanned a number of methods, either paid or free of cost (Table 1). An early partnership with parkrun [18], an organizer of free weekly running events, returned numerous participants through communications in newsletters, presence at events, and parkrun Facebook pages (Figure 1). Direct contact with runners at popular events, competitions for running apparel, and social media presence accounted for peaks in recruitment rates. However, throughout the second half of 2015, participation rates were very low. In 2016, a survey item was added to assess participants’ self-reported recruitment channel, and a new recruitment campaign was initiated. New methods that incurred a cost comprised advertising in Facebook and running-related websites, presence at a fitness expo, and presence at 9 running-related events, including 7 races and 2 Running Film Festival premieres. Facebook paid advertising was conducted with evolving criteria—people interested in running aged 30 to 50 years from February to April and aged 18 to 50 years from May to October. Free strategies comprised the creation of a study Facebook page with regular updates, Web-based media articles and interviews, and agreements with sports-related businesses to provide online discount codes (10%-25% off).

**Figure 2:**
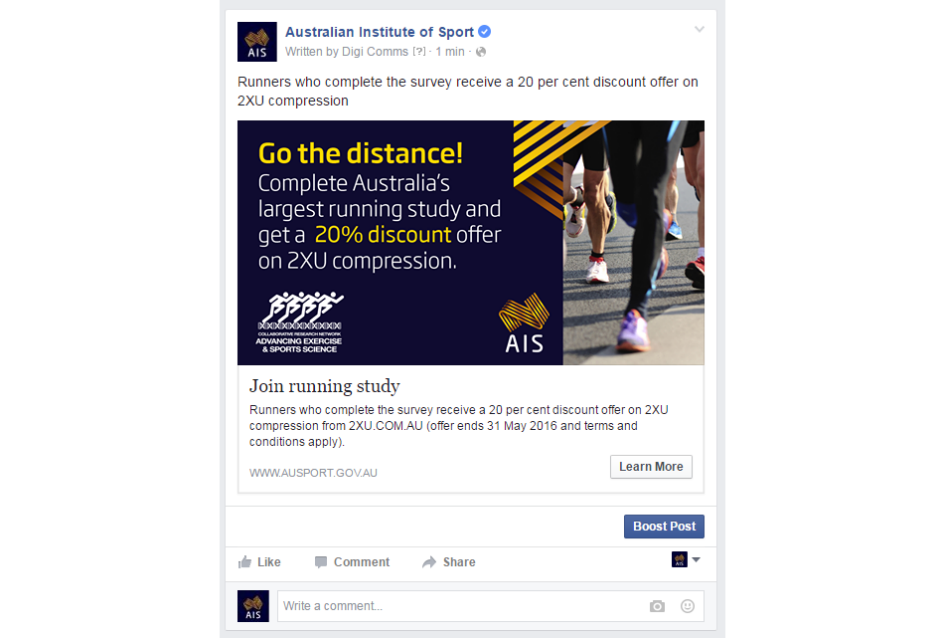
Example of Facebook advertisement including promotional code incentive.

References to incentives were included in the Facebook advertisements (Figure 2). In June 2016, an email campaign contacted all previous respondents (3000 at that stage) requesting help by word of mouth and social media, and hundreds of emails were sent to running event organizers, running clubs, and other running-related businesses. Throughout the recruitment period, the study was supported by the social media, communications, design and commercial capability of the AIS, which provided expertise and some assistance at no cost. The average cost per participant for the period between January and October 2016, excluding the cost of researchers’ time, was estimated as Aus $4 (Table 1). The age and sex representativeness of this cohort could be assessed by comparing these data to AusPlay, the national survey of sports participation [16]. The population estimates from AusPlay were used to derive expected values, and the significance of the chi-square test suggests that the sample selected for our study was overrepresented in runners aged 35 years and older and female runners (Table 2). It must be noted, however, that AusPlay reports participation at least once per year in athletics, track and field, which for over 95% of this population involves running and jogging, and our inclusion criteria required regular running.

Facebook recruited the largest number of participants, but it was also the most diverse strategy, comprising paid and free channels such as targeted advertising, our study page, posts on running-related pages, and personal referral. Other strategies contributed directly to recruitment and frequently provided additional outcomes, for example physical presence at events allowed contact with business representatives which led to promo codes or contact with bloggers or journalists which in turn led to interviews and articles. Some of these additional outcomes ultimately returned more participants than a direct contact with runners.

Different recruitment methods delivered different demographic associations (see Multimedia Appendix 1). The standardized residuals of the chi-square statistic suggested a high contribution of 35- to 44-year-old participants to recruitment by Facebook. Older participants were overrepresented when recruitment was mediated by other social media and parkrun, and participants recruited through the AIS tended to be overrepresented in the 18 to 24 age category. Facebook recruitment showed an association with female participation, whereas Web-based media (blogs, podcasts, and newsletters) appeared to recruit more male participants. People whose country of citizenship is not Australia were less likely to be recruited by Web-based media but more easily recruited through direct presence at running events.

**Table 2:** Age and sex estimates on athletics, track and field participation from the 2016 AusPlay telephonic survey. Expected values were derived from the AusPlay estimates and tested against observed values.

Age and sex categories		AusPlay survey Estimate (×1000)	This study		Significance
			Expected	Observed	Chi-squared *P* value
Total		2891.3	4976	4976	
**Age in years**					
	18-24	567.9	977	356^a^	
	25-34	897.3	1544	1176^a^	
	35-44	713.4	1228	1635^a^	
	45-54	466.9	804	1226^a^	
	55-64	174.5	300	463^a^	
	65 and older	71.3	123	120	<.001
**Sex**					
	Male	1618.4	2785	2272^a^	
	Female	1273.0	2191	2704^a^	<.001

^a^Cells that are the main contributors to the chi-square test statistic.

Respondents reported their average weekly running distance (see Multimedia Appendix 1), and the chi-square test revealed a significant association between recruitment method and distance covered. The standardized residuals showed that participants who run less than 20 km per week were more highly represented in the Facebook and parkrun categories. In contrast, those who run 40 km per week or more, showed the least presence in the parkrun category. As the study was focused specifically on injury, this factor was also tested for association with recruitment methods. Over half of the participants had sustained an injury in the past 2 years, however this rate was not significantly different between the different recruitment categories. No association was found between ethnic origin or eligibility for genetic analysis and recruitment method. Overall, recruitment methods appear to have affected runners’ profiles with regard to age, sex, country of citizenship, and weekly running distance, and the overall sample distribution is dominated by older respondents.

### Aim 2: The Genetic Study

Age is associated with respondents’ attitudes and actual participation in genetic analysis. Early in the survey, before exposure to demographic questions and after exposure to the participant information sheet, participants had the option to tick a box for each of the following items: “I give permission, if I am eligible, to be contacted in the future for related research” and “I give permission, if I am eligible, to be contacted in the future to provide a saliva sample for genetic-related analysis.” Positive answers were given, respectively, by 93.91% (4663/4965) and 89.81% (4459/4965) of participants (Table 3). The chi-square test showed significance for both items, with the 18- to 24-year-old group as the largest contributor to the former, and both age groups below 35 as key contributors to the latter item. Overall, the rates of positive answers tended to increase steadily with older age, from 75.1% (266/354) to 96.6% (115/119) for willingness to participate in genetic research. The association with sex was weak and potentially confounded by an association between age and sex. Participants were also asked the ethnic group of each of their grandparents because an exclusion criterion for the genetic study was to have less than 3 Caucasian European or Mediterranean grandparents. The results suggest that this majority group was more willing to participate in genetic or other research than the minority group, formed by individuals with 2 or more grandparents of Indigenous Australian or Torres Strait Islander, Polynesian, Asian, African, other, or unknown ethnicity.

**Figure 3:**
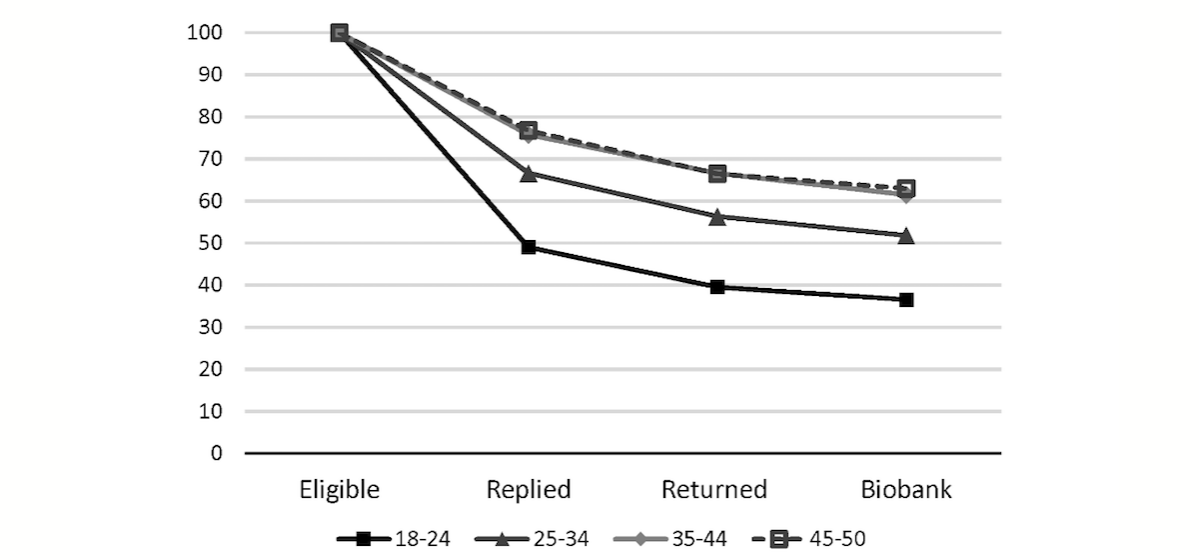
Percentage response decline throughout the genetic arm of the study, from eligibility (including stated willingness to participate) to reply to email requesting contact details (“Replied”), sample return by mail (“Returned”) and biobank consent, categorised by age groups. The most significant decline was seen in the reply rate of the 18-24 year old participants.

A total of 1664 participants met the inclusion criteria for genetic analysis, including consent, ethnicity, and age, and the following results are limited to this cohort (See Multimedia Appendix 2). Eligible participants were contacted by email to request confirmation of contact details. Of all the participants 9 lived overseas, 4 declined participation at this stage, and 278 were deemed unreachable. A total of 1323 out of 1664 (80%) participants replied to emails requesting contact details and 1142 of 1323 (86%) returned saliva samples. Younger age was significantly associated with a lower rate of response to emails requesting contact details, but it did not affect sample return rates. A total of 1038 of 1142 participants (91%) consented for sample storage and biobanking, and no association was observed with age or sex. Overall, no independent association was found between recruitment strategy and follow-up rate, sample return, or biobank consent (results not shown). A graph depicting the rates of participants’ loss in the different age brackets throughout the study is shown in Figure 3.

**Table 3:** Willingness to be contacted for related or genetic research as stated by respondents in the survey, shown by ethnicity, age or sex.

Categories		Total (n=4965)	Permission to be contacted for related research			Permission to be contacted for genetic analysis		
			Yes (n=4663)	No (n=302)	Chi-squared *P* value	Yes (n=4459)	No (n=506)	Chi-squared *P* value
**Ethnicity, n (%)^a^**					<.001			<.001
	Caucasian European/ Mediterranean	4508 (87.8)	4255 (94.39)	253 (5.61)		4090 (90.73)	418 (9.27)	
	Other	456 (5.0)	410 (89.9)	46 (10.1)^a^		371 (81.4)	85 (18.6)^a^	
**Age, n (%)**					<.001			<.001
	18-24	354 (7.1)	309 (87.3)	45 (12.7)^b^		267 (75.4)	87 (24.6)^b^	
	25-34	1173 (23.6)	1076 (91.73)	97 (8.27)		1012 (86.27)	161 (13.73)^b^	
	35-44	1632 (32.9)	1554 (95.22)	78 (4.78)		1495 (91.61)	137 (8.39)	
	45-54	1225 (24.7)	1165 (95.10)	60 (4.90)		1139 (92.98)	86 (7.02)	
	55-64	462 (9.3)	444 (96.1)	18 (3.9)		430 (93.1)	32 (6.9)	
	65 and over	119 (2.4)	115 (96.6)	4 (3.4)		116 (97.5)	3 (2.5)	
**Sex, n (%)**					.499			<.01
	Male	2266	2122 (93.65)	144 (6.35)		2063 (91.04)	203 (8.96)	
	Female	2699	2541 (94.15)	158 (5.85)		2396 (88.77)	303 (11.23)	

^a^Missing n=1.

^b^Cells that are the main contributors to the chi-square test statistic.

## Discussion

### Part 1: The Recruitment Process

Web-based health data collection is now possible because of the ubiquitous presence of the internet and the multiplicity of access devices. In addition, technologies for the collection of saliva samples without a skilled practitioner or refrigeration in transit facilitate remote donations for genetic analysis. These two advances enabled the design of an epidemiological and genetic study with the ambition of recruiting 10,000 survey participants and obtain at least 3000 genetic samples [14]. The final numbers, however, amounted to slightly under 5000 participants and 1142 samples in a period of 2 years [13]. This sample size leaves the genetic study underpowered, but with potential to contribute to meta-analyses and replication studies. The number of adult runners in Australia in 2015-2016 was estimated over 2.8 million [16], so there was no shortage of potential participants. Here we analyzed the recruitment strategies employed, their efficiency, and their impact on population representativeness. We also searched for associations between participants’ characteristics, recruitment, and retention throughout the genetic arm of the study.

Every recruitment method shows potential for bias [19], so to minimize overall bias we used a combination of recruitment strategies. Nevertheless, a comparison with data from the randomly sampled AusPlay sport participation survey [16] revealed a significant underrepresentation of young adults. It must be noted that the analysis of recruitment strategies presented here is based on data obtained in the second wave of recruitment, after an item addressing the source of recruitment was included in the survey, and provides no information on recruitment trends in the initial wave of recruitment. Despite social media being considered as a suitable platform for this population [20] and Facebook being the most popular social media platform in Australia for all ages [21], neither Facebook nor other social media boosted the participation of younger adults in our study. Facebook preferentially recruited participants in the 35 to 44 years age bracket, which may be a result of our advertising campaign being targeted to runners aged 30 to 50 years during the first 3 months, before it was extended to ages 18 to 50 years for another 6 months. However, targeted advertising was only one of the many Facebook-related strategies used to promote the study, in addition to snow-balling, presence on pages for running groups, and others. It is possible that physically active young adults are just difficult to reach, and use of Facebook is not sufficient to promote a research study to this population. Alternative channels such as universities or sports organizations may have been more successful. In fact, our own website, the AIS, which provides programs and facilities for elite athletes, was the only channel that recruited a representative proportion of young adults. Conversely, a network of free, weekly, timed 5 km outdoor runs called parkrun recruited numerous participants, but a high proportion of those were in the 55 to 64 years age category. Overall, the reported bias in age distribution for each recruitment method highlights the need to use a range of recruitment activities. One cannot expect, however, that a mix of methods will cancel bias, as is the case with this study where the most successful strategies returned samples biased toward older ages.

Other associations were evident. Sex distribution was affected by Facebook and Web-based media. Despite an absence of sex-related differences in social media or Facebook usage or behavior [21], the female recruitment rate through Facebook of 61.4% (601/979) was high, in line with previous studies that also used Facebook (60% in average) [5]. The large contribution of the Facebook cohort to the sample may have been responsible for the overall female predominance in the study, although it is not unusual for women to show higher rates of enrollment in health research studies [22]. Respondents who run less than 20 km per week were overrepresented and those who run over 40 km per week were underrepresented in the parkrun group, which aligns with the observation that parkrun attracts people with lower running ability [23].

Overall, no specific strategy combined the desired requirements for high recruitment rate, population representativeness, and low cost; however the relative representation reported here could help researchers choose the recruitment methods that best fit their target population. Additionally, it is recommended that an item asking for participants’ self-reported method of recruitment is included in surveys so that these data can be used to assess or modify recruitment strategies. This study was characterized by two waves of intensified recruitment, one at study roll-out and another after a long lag in recruitment. Each wave involved consultation with the AIS communications team and the design of a tailored recruitment plan. The initial campaign was successful but it lacked momentum, and another campaign, reported here in detail, was required to boost recruitment. Despite the assistance of communication experts and the use of a reputable and popular brand such as the AIS, the study only recruited half of its projected participants. According to our experience, large-scale recruitment for internet data collection sits at the interface of marketing and research and acts in a direct competition with many other scientific studies and market research. In this competitive environment, the communication requirements for large-scale recruitment are beyond the researchers’ expertise. Only by engaging marketing experts and allocating sufficient resources will a study succeed in large-scale recruiting.

### Part 2: Respondent Participation in the Genetic Study

Significant time and consideration was invested in developing a process of informed consent that explained the study’s purpose in a clear and simple way. The participant information sheet was followed by requests for permission to be contacted for related research and for genetic analysis. The fact that the former question, open and nonspecific, received a slightly higher positive answer rate than the latter, which is linked to this specific research study and backed by detailed information, aligns with reports indicating that the presence of a genetic component in studies aimed at the general population has a negative impact on survey participation [10,24-26]. Overall, willingness to participate in the genetic arm of the study was slightly higher than in other studies (90% vs 83-86%) [10,26,27]. As shown by others, younger age was associated with a negative answer to both requests [11,26,27]. This could be linked to lower levels of trust in research and greater privacy concerns in younger adults [11,26,28], and a more altruistic attitude to research in older adults [29]. Those who were not Caucasian European or Mediterranean were significantly more likely to answer negatively despite the survey item for ethnicity being located well after the request for permission to be contacted. This agrees with some studies examining the willingness of ethnic minorities to participate in genetic research [3,22,26] but not with others [4,27]. It must be noted that all these studies were conducted in the United States and the minorities described were African American and Hispanic, whereas our study was conducted in Australia and the minorities encountered were primarily Asian, Indigenous Australian, Pacific Islander, and African. The combined observations indicate that there is a broader pattern suggesting that recruitment should be tailored to enhance representation of ethnic minorities in genetic studies.

Only 80% of eligible, willing participants responded to the follow-up email, similarly to previous genetic research studies [3,4]. We can only speculate about the reasons for this drop. As people’s belief in the importance of genetic research appears to be higher than their individual willingness to participate [8], it is likely that participants only considered their individual concerns when confronted with actual participation. Alternatively, email, the vehicle of contact, is prone to loss by incorrect records or spam filters, or it may simply be ignored. Accordingly, the fact that response rates approached 65% in the 18 to 24 years age category versus 83% in the 35 to 50 years age category may reflect the abovementioned association between age and attitude to research or privacy, or it could reveal age-related differences in email usage. Previously reported age effects in studies that did not involve email [3], combined with recent data supporting that email is still strong in young adults [30], seem to indicate that the effect stems from different attitudes to research or privacy. We recommend that studies targeting young adults design contact protocols that match the specific attitudes supported by this population.

An additional drop in retention occurred when participants were mailed the materials needed to donate their sample. Despite the simple procedure designed to minimize participant burden, only 86% of the samples were returned. However others have reported lower saliva kit return rates between 42% and 82% [2,3,31,32]. Unlike another study [3], no age associations were found at this stage, although the older age categories tended to show higher return rates. This was the most expensive drop in participation because it combined administrative costs, postal fees, and the cost of the collection kits. Informal feedback from participants indicated that they might have been more engaged had they been given a deadline for sample return. A study in young adults (age around 28 years) found that offering a small incentive for sample collection tripled the odds of obtaining a sample but decreased the likelihood of obtaining biobank consent [4]. These strategies were not tested by us but may be worth considering. Of those respondents who returned the saliva sample, 93% gave consent for biobank storage, irrespective of age or sex. This rate reveals that, once enrolled in the study and having donated a sample, participants generally see the benefits of storage and sharing their deidentified sample for future studies.

Overall, this analysis reveals an age-related bias experienced throughout the study. Sex was associated with survey participation but not with other steps of the process, in agreement with some studies [27] but not with others [3,22,33]. Our sample comprised physically active people and participation rates suggest that they are at least equivalent to other populations in their likelihood to participate in genetics research. There are two previous studies providing evidence for [3] or against [22] an association between physical activity, or exercise capacity, and participation in genetics studies in participants aged 40 years or older. Nonetheless, to our knowledge, no studies to date have examined this kind of association in younger cohorts, and our data may suggest that physically active people aged under 35 years are particularly hard to reach and retain for genetics research. Therefore, it is recommended that studies targeted at this population use recruitment strategies designed to match the specific characteristics of this population.

This study is not without limitations. It is an observational study that lacks a systematic assessment of confounding factors. Recruitment strategies were self-reported, and our measure of recruitment strategies from the participants’ response to the survey item is a gross measure. In addition, some recruitment channels, such as promotions and the email campaign to previous respondents, could not be assessed for success because these items were not included in this survey item. This item was added to the survey over halfway through the recruitment period, precluding any analysis on the initial phase of recruitment. The target population for Facebook advertising, a successful recruitment channel in this study, was also updated halfway through the campaign. This adaptive move, which aided with recruitment to satisfy the primary aims of the study, imposes an additional limitation to the current analysis. A limitation to the generalizability of the results is the inclusion or exclusion criteria of both the survey-based and genetic arms of the study.

### Conclusions

The internet undoubtedly offers many opportunities to reach potential participants for genetic research. However, the results of this study show that initial contact and follow-up methods need to be designed according to the target population. A caveat of these studies is that, even though initial recruitment is done through popular channels such as social media, subsequent contact needs to be done in person or by mail, which exposes these studies to the traditional hurdles such as email or parcel loss, change of address or loss of participants’ interest due to lack of immediacy. Contact by email could be replaced by more immediate channels such as text message or social media; however, the requirement for a mailing address for parcel delivery will continue to be a challenge for recruitment, and it is difficult to conceive a system that would eliminate this challenge.

## Appendix

Multimedia Appendix 1 Respondents’ characteristics by recruitment strategy. Superscript "a" indicates cells that are the main contributors to the chi-square test statistic.

Multimedia Appendix 2 Participants who replied to contact emails for the genetic arm of the study, those who returned the sample and those who provided signed consent to biobank their sample, calculated as percentages of the previous category and categorized by age and sex. Superscript "a" indicates cells that are the main contributors to the χ2 test statistic.

## Abbreviations

AIS: Australian Institute of Sport
